# Trimethylamine *N*-Oxide: The Good, the Bad and the Unknown

**DOI:** 10.3390/toxins8110326

**Published:** 2016-11-08

**Authors:** Manuel T. Velasquez, Ali Ramezani, Alotaibi Manal, Dominic S. Raj

**Affiliations:** Division of Renal Diseases and Hypertension, The George Washington University School of Medicine, 2150 Pennsylvania Avenue NW, Washington, DC 20037, USA; mvelasquez@mfa.gwu.edu (M.T.V.); ramezani@gwu.edu (A.R.); malotaibi@email.gwu.edu (A.M.)

**Keywords:** microbiome, dysbiosis, uremic toxin, cardiovascular disease

## Abstract

Trimethylamine *N*-oxide (TMAO) is a small colorless amine oxide generated from choline, betaine, and carnitine by gut microbial metabolism. It accumulates in the tissue of marine animals in high concentrations and protects against the protein-destabilizing effects of urea. Plasma level of TMAO is determined by a number of factors including diet, gut microbial flora and liver flavin monooxygenase activity. In humans, a positive correlation between elevated plasma levels of TMAO and an increased risk for major adverse cardiovascular events and death is reported. The atherogenic effect of TMAO is attributed to alterations in cholesterol and bile acid metabolism, activation of inflammatory pathways and promotion foam cell formation. TMAO levels increase with decreasing levels of kidney function and is associated with mortality in patients with chronic kidney disease. A number of therapeutic strategies are being explored to reduce TMAO levels, including use of oral broad spectrum antibiotics, promoting the growth of bacteria that utilize TMAO as substrate and the development of target-specific molecules with varying level of success. Despite the accumulating evidence, it is questioned whether TMAO is the mediator of a bystander in the disease process. Thus, it is important to undertake studies examining the cellular signaling in physiology and pathological states in order to establish the role of TMAO in health and disease in humans.

## 1. Introduction

Trimethylamine *N*-oxide (TMAO) is a small organic compound in the class of amine oxides with a molecular mass 75.1 Daltons. It is frequently found in the tissues of a variety of marine organisms [1] including marine elasmobranch, in which TMAO is known to protect against the adverse effects of temperature, salinity, high urea and hydrostatic pressure. For instance, urea destabilizes many macromolecular structures and inhibits cellular functions such as ligand binding, which is counteracted by TMAO [2]. TMAO can be metabolized to small methylated amines such as tri-, di-, and monomethylamine, which are precursors of marine aerosols and the greenhouse gas nitrous oxide in the marine atmosphere. Although known to marine biologists for a long time, TMAO came into the limelight recently with the reported association with cardiovascular disease in humans [3].

In organisms that concentrate urea as an osmolyte and buoyancy factor, TMAO has been shown to restore proteins to their native structure and allow them to regain their enzyme function that has been lost because of the presence of urea [4,5]. Furthermore, in deep see creatures, TMAO, and, to a lesser extent, betaine, counteract the inhibitory effects of hydrostatic pressure on proteins [6]. Although it is widely known that TMAO stabilizes the folded state of proteins, the mechanism by which TMAO interacts with proteins remains speculative. One hypothesis is that the amphiphilic structural arrangement of TMAO allows it to form H bonds with water and preferentially exclude or interact with certain protein functional groups [7].

## 2. Biosynthesis of TMAO

TMAO is formed from trimethylamine (TMA), which is generated by the action of gut microbiota from dietary choline and phosphatidylcholine (lecithin) [8,9]. Koeth et al. described another pathway which involves the catabolism of l-carnitine through two sequential microbial reactions. According to this, l-carnitine is first converted into the intermediate metabolite γ-butyrobetaine and then into TMA, which is subsequently converted into TMAO by host hepatic flavin monooxygenases 3 (FMO3) [10]. Red meat, eggs, dairy products and salt-water fish are rich in choline, lecithin, and carnitine and, hence, are a potential source of TMAO. TMA is a gas which is oxygenated within living animals to form TMAO by flavin monooxygenases (FMO1 and FMO3) [11,12]. TMAO is then either transported to the tissues for accumulation as an osmolyte or, more commonly, cleared by the kidney [3,9,13,14]. In humans, a mutation of the FMO3 causes trimethylaminuria, a condition wherein individuals excrete TMA, rather than TMAO. This condition known as fish odor syndrome is characterized by a fishy body odor in urine, sweat, breath and other bodily excretions [15]. 

## 3. Role of Microbiome in TMAO Generation 

TMAO is commonly found in a variety of marine microbiota, where it serves as an important substrate in the anaerobic metabolism of a number of bacteria [16]. Studies in gnotobiotic mice have shown that TMAO accumulates in the serum of animals colonized with TMA-producing bacteria, but not in animals colonized with bacteria that do not generate TMA from choline in vitro [17]. Further evidence that the production of TMAO is dependent on metabolism by intestinal microbiota is provided by studies in healthy humans given phosphatidylcholine challenge, in which the plasma levels of TMAO were suppressed by oral administration of broad spectrum antibiotics [14].

Human and animal studies suggest that several families of bacteria are involved in TMA/TMAO production, namely, Deferribacteraceae, Anaeroplasmataceae, Prevotellaceae [3] and Enterobacteriaceae [18,19]. Recent studies using isolates of commensal bacteria in the human intestine have identified nine strains capable of producing TMA from choline in vitro: eight species representing two different phyla (*Firmicutes* and *Proteobacteria*) and six genera that showed significant choline consumption and TMA production including *Anaerococcus hydrogenalis*, *Clostridium asparagiforme*, *C. hathewayi*, *C. sporogenes*, *Escherichia fergusonii*, *Proteus penneri*, *Providencia rettgeri*, and *Edwardsiella tarda* [17]. Recently, experiments with the choline-degrading sulfate reducing bacterium *Desulfovibrio desulfuricans* identified a gene cluster (including cutD) involved in radical C–N bond cleavage of choline leading to generation of TMA [18].

## 4. Determinants of TMAO Level 

The plasma TMAO levels show wide inter- and intra-individual variations. Circulating levels of TMAO are determined by a number of factors, including diet, gut microbial flora, liver FMO enzymes and kidney function [17]. Heritability of TMAO was studied in a genome-wide association study involving 1973 humans, which concluded that genes play only a marginal role in determining TMAO levels [20]. Wang et al. measured fasting plasma levels of TMAO in 349 apparently healthy subjects using stable isotope dilution liquid chromatography tandem mass spectrometry (LC/MS/MS) and reported a median concentration of 3.45 μM (Interquartile Range 2.25–5.79), which did not differ by sex, but the levels increased with advancing age [21]. Kühn et al. measured fasting plasma levels of TMAO at two time points in the European Investigation into Cancer and Nutrition (EPIC)-Heidelberg study participants and noted a significant intra-individual variation in TMAO levels [22]. In a crossover feeding trial, consumption of diet containing TMAO precursors such as eggs, beef and fish increased the blood and urine levels of TMAO [23]. Circulating TMAO concentrations in response to the fish meal was increased within 15 min of food consumption, suggesting that TMAO itself can be absorbed without undergoing processing by the gut microbes [23]. However, not all studies found an association between diet and TMAO levels [22]. In a study involving 271 participants, consumption of meat, egg, or fish was not associated with TMAO, choline, or betaine concentrations [24]. 

Studies on pharmacokinetics [25] and renal clearance of TMAO [26] in healthy human subjects suggest that TMAO has a small volume of distribution—about one half that of urea—but a higher renal clearance compared to urea and creatinine. In normal subjects, the urinary clearance of TMAO was 219 ± 78 mL/min compared to the urinary urea and creatinine clearances 55 ± 14 and 119 ± 21 mL/min, respectively. The high renal clearance rate of TMAO indicates that in addition to glomerular filtration, at least 50% of its renal excretion possibly occurs through tubular secretion. 

Missailidis et al. measured plasma concentration of TMAO in 80 controls and 179 CKD patients and reported that that elevated TMAO levels are strongly associated with degree of renal function [27]. Kaysen et al. measured serum levels of TMAO in 235 hemodialysis patients and reported that serum TMAO concentrations (median 43, (25th–75th percentile 28–67 μM/L)) were elevated in these patients compared to persons with normal or near normal kidney function (1.41 ± 0.49 μM/L) [8]. Bain et al. noted that the concentrations of TMA and TMAO in pre-dialysis plasma (1.39 ± 0.483 and 99.9 ± 31.9 mM, respectively) were significantly higher than the corresponding levels in healthy subjects (0.418 ± 0.124 and 37.8 ± 20.4 mM, respectively) [28]. There was a significant reduction (about 60%) in plasma TMA and TMAO during a single hemodialysis session and the post-dialysis levels were not significantly different from that in control subjects. As expected, successful kidney transplantation resulted in substantial reductions in TMAO concentrations [29].

## 5. TMAO and Cardiovascular Disease

The concept of the meta-organism was first proposed by an insightful German zoologist, Karl Möbius, to describe the inter-dependency between animal species [30]. To date, a number of meta-organismal metabolic pathways involving interactions between the gut microbiome and the human host have been discovered. Indeed, TMAO generated by gut microbiome exacerbates impaired glucose tolerance, inhibits hepatic insulin signaling, and promotes adipose tissue inflammation in mice that are maintained on a high-fat high-sugar diet [17]. In animals and humans, TMAO has also been suggested as a strong candidate molecule mediating the development of type-2 diabetes mellitus [31]. Using a metabolomics approach, Wang et al. discovered a cluster of three phospholipid-associated molecules choline, betaine, and TMAO, associated with atherosclerosis [32]. Further studies in a murine model of atherosclerosis demonstrated that plasma TMAO levels in apoE^−/−^ mice positively correlated with atheroma burden [32]. Choline diet increased the foam cell formation with accompanying increase in scavenger receptor CD36 and SRA1 protein in murine macrophages. In 3903 sequential stable subjects undergoing elective diagnostic coronary angiography, elevated plasma levels of choline and betaine were each associated with poor prognosis even after adjusting for traditional cardiovascular risk factors. However, high choline and betaine levels are associated with higher risk of future major cardiac events only with concomitant increased levels of TMAO [33]. In another study, TMAO levels were increased in patients with stable heart failure and elevated levels were associated with increased risk of death [34]. Thus, substantial evidence indicates that elevated TMAO levels are associated with cardiovascular disease (CVD).

Not all studies have demonstrated an association between TMAO and atherosclerotic vascular disease. Mueller et al. measured plasma concentrations of TMAO, betaine and choline in a cohort of 339 patients undergoing coronary angiography and noted that plasma concentrations of TMAO were higher in diabetics compared to euglycemic patients [35]. The plasma levels of TMAO or betaine were not found to be associated with a history of myocardial infarction, the angiographically-assessed presence of coronary heart disease, or the incident cardiovascular events during 8 years of follow-up. In a case-control study of patients with large-artery atherosclerotic ischemic stroke and transient ischemic attack, participants with asymptomatic atherosclerosis did not exhibit an obvious change in gut microbiota and blood TMAO levels; however, stroke and transient ischemic attack patients showed significant dysbiosis of the gut microbiota, and their blood TMAO levels were decreased [36]. 

Although elevated plasma levels of TMAO have been associated with atherosclerotic vascular diseases and increased risk of adverse cardiovascular events, seafood, which contains high levels of TMAO, is generally considered beneficial for health. Another contradictory finding was reported by Bjørnda et al. who demonstrated that feeding male Wistar rats a phospholipid-protein complex (PPC) from Antarctic krill increased the plasma levels of TMAO, choline, and carnitine. However, there was no correlation between plasma choline and TMAO in rats fed PPC [37]. 

## 6. Mechanism of TMAO Associated Atherogenesis

**Figure 1:**
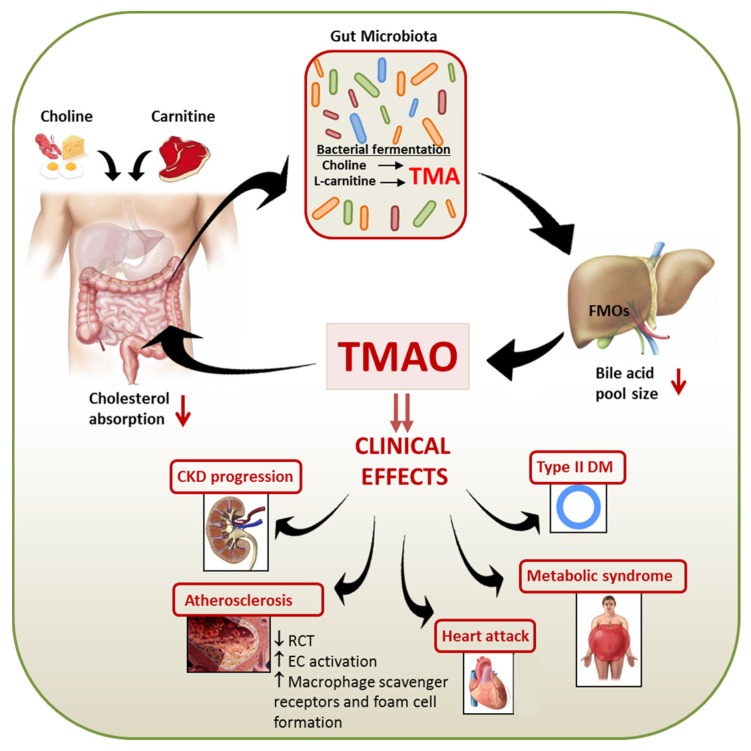
Schematic representation of the link between diet, gut microbiota, TMAO and the clinical manifestations of this uremic toxin. Both host and gut microbial metabolism of trimethylammonium-containing nutrients (e.g., choline, phosphatidylcholine, and l-carnitine) result in the formation of TMA. In liver, FMOs convert TMA into TMAO. Clinical effects of TMAO includes alteration of cholesterol and sterol metabolism, progression of CKD, atherosclerosis, heart attack, metabolic syndrome, type II DM, and alternations in bile acid metabolism and sterol transporters both within the liver and intestine. TMA, Trimethylamine; TMAO, Trimethylamine *N*-Oxide; FMOs, flavin-containing monooxygenases.

The mechanism by which TMAO promotes atherosclerosis also remains speculative. The proposed mechanisms include changes in cholesterol and sterol metabolism, promotion of foam cell formation by increasing expression of scavenger receptors on macrophages, and causing alternations in bile acid metabolism and sterol transporters in the liver and intestine (Figure 1). 

Wang et al. showed that diet containing choline, TMAO or betaine could enhance up-regulation of multiple macrophage scavenger receptors that are linked to atherosclerosis [32,38]. In mice with an intact intestinal microbiota, dietary supplementation with TMAO, carnitine or choline reduced in vivo reverse cholesterol transport [3]. Interestingly, dietary supplementation with TMAO markedly reduces the expression of intestinal cholesterol transporters Niemann-Pick C1-like1 (Npc1L1), and Abcg5/8, which transport cholesterol into and out of enterocytes, respectively [3]. However, the levels of cholesterol transporters in macrophages did not change significantly following TMAO treatment [3]. Also, mice supplemented with dietary TMAO showed a significant reduction in cholesterol absorption, as well as liver expression of bile acid synthetic enzymes cytochrome P450, family 7, subfamily a, polypeptide 1 (Cyp7a1) and cytochrome P450, family 27, subfamily a, polypeptide 1 (Cyp27a1) [3]. The bile acid pathway plays a major role in cholesterol elimination and blocking this pathway may promote atherogenesis. The liver’s levels of bile acid synthetic enzymes (Cyp7a1 and Cyp27a1) and bile acid transporters (Oatp1, Oatp4, Mrp2, and Ntcp) were reduced in mice that were treated with TMAO [3]. 

Both in vivo studies in mice and in vitro studies in cultured human aortic endothelial and vascular smooth muscle cells have shown that physiological levels of TMAO induce expression of inflammatory cytokines and adhesion molecules. This activation was mediated, in part, by the NF-κB signaling pathway [39]. In a series of studies in animals employing dietary choline, or TMAO, germ-free mice, and microbial transplantation, Zhu and colleagues [40] showed that gut microbe-derived TMAO enhances platelet responsiveness and thrombosis in vivo. In addition, studies in humans also showed that plasma TMAO levels independently predicted incident thrombosis (heart attack, stroke) risk [40]. Direct exposure of platelets to TMAO enhanced sub-maximal stimulus-dependent platelet activation from multiple agonists through augmented Ca^2+^ release from intracellular stores. Collectively, these findings provide evidence for a mechanistic link between gut microbes, platelet function, and thrombosis risk [40]. On the contrary, Collins et al. showed that TMAO at concentrations up to 10-fold the Cmax reported in humans did not affect in vitro foam cell formation in apoE^−/−^ mice transfected with human cholesteryl ester transfer protein [41]. Surprisingly, TMAO levels were inversely correlated with atherosclerotic lesion size in aorta. This study suggests that TMAO may confer a protective and not a causative effect on atherosclerosis development. Thus, further studies are needed to conclusively prove the role of TMAO in the pathogenesis of atherosclerosis. 

## 7. TMAO in Chronic Kidney Disease 

Uremic toxins are organic solutes normally metabolized and excreted by the kidneys, but they often accumulate in the presence of impaired kidney function, causing toxicity. They can be classified based on their size and clearance by dialysis, as well as their source such as food, endogenous metabolism and gut microbiota [42]. Gut microbial profile is altered in patients with chronic kidney disease (CKD). Dysbiosis in CKD has been touted to be due to slow intestinal transit time, delivery of undigested protein to the colon, decreased dietary fiber intake, iron therapy, frequent use of proton pump inhibitors and antibiotics [43]. Vaziri et al. noted that 190 microbial operational taxonomic units differed significantly in abundance between patients with end-stage renal disease and apparently healthy controls. Abundant evidence indicates that gut microbiome is the source of a number of retention solutes in patients with CKD [43]. 

TMAO and choline levels increase with decreasing renal function [35]. However, it is unclear whether dysbiosis leads to increased generation of TMAO in CKD. Hai et al. showed that the production rate of TMAO is not higher in end-stage renal disease (ESRD) patients compared to control subjects [26]. Under normal physiologic conditions, circulating TMAO is rapidly cleared almost exclusively by urinary excretion [44]. Niwa et al. proposed the protein metabolite hypothesis that endogenous protein metabolites play a significant role in the progression of CKD [45]. Tang et al. showed that C57BL/6J mice fed with choline or TMAO exhibited tubulointerstitital fibrosis, collagen deposition and phosphorylation of Smad3; an important regulator of the profibrotic transforming growth factor-β/Smad3 signaling pathway [9]. A prospective cohort study of 521 stable subjects with CKD found elevated plasma TMAO levels which were associated with a 2.8-fold increased mortality risk [9]. Stubbs et al. showed that TMAO concentration is an independent predictor for coronary atherosclerosis burden and predicted long-term mortality independent of traditional cardiac risk factors [29]. TMAO concentrations in ESRD patients on dialysis are 40-fold higher compared with control subjects [26]. Fortunately, the small volume of distribution enables adequate clearance by dialysis. In HEMO study participants, higher TMAO concentrations were associated with a higher risk of sudden cardiac death, first cardiovascular event, and any-cause death, particularly in whites [46]. A cross-sectional study by Aki Mafune et al. observed elevated TMAO levels in CKD patients who underwent cardiovascular surgery (*n* = 227) [47]. Patients with the highest levels of TMAO had a higher number of infarcted coronary arteries compared to those with the lowest levels [47]. On the other hand, Kaysen et al., did not find any significant association between serum TMAO concentrations and all-cause mortality, cardiovascular death, or hospitalizations in 235 hemodialysis patients [8]. Thus, it remains unclear whether elevated TMAO levels in CKD are due to increased generation or decreased elimination, and whether it is just a surrogate for other retention solutes. 

## 8. TMAO: Toxin, Marker or Bystander?

Emerging evidence indicates that consumption of diet high in carnitine or rich in choline could potentially lead to increased cardiovascular disease through generation of TMAO [3]. The Atherosclerosis Risk in Communities study [48] and the European Prospective Investigation into Cancer and Nutrition study [49] did not report increased cardiovascular risk with increasing dietary intake of choline. In addition, fish, which is an important source of trimethylamine in the diet [50], is not associated with risk of cardiovascular disease in the Physicians’ Health Study [51]. On the contrary, a meta-analysis concluded that fish consumption is inversely associated with fatal coronary heart disease [52].

Sharks must continually balance their internal osmotic pressure with that of the external marine environment by accumulating urea and TMAO as osmotic agents. The latter counters the protein-destabilizing effects of urea and thus has beneficial effect. However, concentrated TMAO is responsible for the toxicity of fresh Greenland Shark (*Somniosus microcephalus*) meat. On digestion, TMAO breaks down to trimethylamine, which induces severe gastrointestinal and neurological effects similar to extreme drunkenness. It is unknown whether chronic consumption of Greenland Shark meat will increase the risk of cardiovascular disease. It is possible that the marine animals have developed adaptive mechanisms to TMAO, which is poorly developed in humans. 

## 9. Therapeutic Strategies

Several lines of evidence indicate that the gut microbiota plays a role in systemic inflammation, metabolic syndrome, vascular dysfunction and atherosclerosis. Thus, investigators have explored the possibility of modulating gut microbes and their metabolites for health benefits. However, such efforts have been hampered by the complexity of the interaction of microbiome with host genetics, host diet, and other unknown factors. The discovery of TMAO as a potential gut derived toxin with cardiovascular disease has energized such efforts. A number of therapeutic strategies have been tested from a broad spectrum of antibiotics to target-specific molecules with varying levels of success. (Table 1).

Assuming the microbiome is the source of TMAO, antibiotic treatment should reduce its circulating levels. Indeed, in health participants, suppression of intestinal microbiota with oral broad spectrum antibiotic reduced plasma TMAO levels after a phosphatidylcholine challenge [14]. TMAO levels increased one month after stopping antibiotics [14]. However, antibiotics is not an ideal treatment since it could have other unwanted consequences, and also chronic treatment is likely to lead to emergence of resistant bacteria. 

Besides removing TMAO producing bacteria, use of selected bacteria to increase its metabolism has also been attempted. TMA could be depleted in vivo by bioconversion into a molecule with no undesirable properties. Brugere et al. [58] proposed the use of archaeobiotics to prevent trimethylaminuria accumulation and cardiovascular disease. This is based on the concept of reducing TMA formation in the gut by converting it into an inert molecule, such as methane [58]. A number of methanogenic bacteria belonging to the order *Methanobacteriales* have been identified as natural inhabitants in the human gut. These archaea utilize only methyl compounds including TMA as substrate [58]. Various bacteria grow anaerobically using TMAO as an alternative terminal electron acceptor of a respiratory transport chain [16]. During this energy-yielding reaction, TMAO is reduced to volatile TMA. The bacteria capable of reducing TMAO to TMA are found in different ecological niches such as the marine environment (Photobacterium, Shewanella and Vibrio species), in photosynthetic bacteria living in ponds (Rhodobacter species), and is also found among many enterobacteria [16,53,54]. Thus, colonizing human gut with such bacteria could reduce TMAO levels, but this concept needs to be further explored. 

Reduced intake of dietary precursors of TMAO is a potential approach. Dietary l-carnitine has been shown to induce gut microbiota-dependent production of TMA and TMAO in an animal study [3]. Thus, decreased consumption of l-carnitine and choline could decrease TMAO levels [3]. A systematic review and meta-analysis of 13 controlled trials showed that l-carnitine is associated with a 27% reduction in all-cause mortality, a 65% reduction in ventricular arrhythmias and a 40% reduction in anginal symptoms in patients experiencing an acute myocardial infarction compared to controls [59]. 

Resveratrol (RSV) is a natural polyphenol with prebiotic benefit that mainly occurs in grapes and berries. Chen et al. examined the effects of RSV on TMAO induced atherosclerosis, gut microbiota and TMAO synthesis in C57BL/6J and apoE^−/−^ mice. Treatment with RSV resulted in an increase in *Bacteroidetes* abundance at the expense of *Firmicutes* in choline-treated apoE^−/−^ mice. Serum TMA and TMAO levels were much lower in RSV-treated mice fed with choline than in those in the control group [55]. 

Meldonium, an aza-analogue of gamma-butyrobetaine (GBB), competes with quaternary amines, including l-carnitine and GBB, for selected enzymes and transport proteins in the body. It decreased intestinal microbiota-dependent production of TMA/TMAO from l-carnitine in Wistar rats [56]. Meldonium, however, did not influence bacterial growth and bacterial uptake of l-carnitine [56].

3,3-dimethyl-1-butanol (DMB) is a structural analog of choline and an inhibitor for TMA formation through inhibition of microbial TMA lyases. DMB is found in some balsamic vinegar, red wines, and in some olive oils and grape seed oils. DMB inhibited choline diet-enhanced endogenous macrophage foam cell formation and atherosclerotic lesion development in apoE^−/−^ mice without alterations in circulating cholesterol levels [38]. Furthermore, DMB promoted reduction in microbial taxa that are associated with plasma TMA and TMAO levels and also reduced the choline-diet-dependent enhancement in aortic root atherosclerotic lesion development [38].

FMO3 is the member of the FMO enzyme family with the highest activity level and is capable of oxidizing TMA to TMAO. A potential site of therapeutic intervention could be at the level of the host enzyme machinery necessary for the conversion of TMA to TMAO. The knockdown of FMO3 using antisense oligonucleotide in mice has been shown to attenuate atherosclerosis [57]. The knockdown of FMO3 not only resulted in decreased TMAO levels but also regulated both lipid metabolism and inflammation, thereby attenuating atherosclerosis. It is important to recall that individuals without functional FMO3 often exhibit a fishy odor, which is caused by trimethylaminuria.

To summarize, recent studies point to the potential contribution of gut microbiota-derived production of TMAO from the metabolism of dietary choline and l-carnitine and the increased risk of developing atherosclerotic heart disease. Interestingly, TMAO plays an important physiological role in lower animals, but in humans it is merely a remnant of the evolution of the osmolyte system. It is possible that accumulation of TMAO in humans in disease state may be an adaptation of cells to stress and, hence, a marker rather than a mediator of disease. On the other hand, there is accumulating clinical and laboratory-based studies attributing a pathogenic role to this molecule. It is important to undertake studies examining intracellular concentrations of TMAO in mammals, cellular signaling and also determine the effects of TMAO on enzymes and other proteins in order to establish the role of TMAO in health and disease in humans. 

## Figures and Tables

**Table 1:** Therapeutic approaches to reducing TMAO concentration.

Therapy	The Effects	Remarks
Antibiotics [14]	Decreased TMAO plasma levels	Nonspecific, chronic use impossible and emergence of antibiotic resistance is likely
Microbiomes [16,53,54]	Decreased TMA formation in the gut	Safety and engraftment unclear in human
	Reducing TMAO to TMA	
Reduced l-carnitine and choline consumption [3]	Decrease TMAO level	l-carnitine is found to reduce all-cause mortality, cardiac symptoms in patients with myocardial infarction
Resveratrol [55]	Decreased TMA and TMAO plasma levels	It also changed the quantities of microbes
Meldonium [56]	Reduced TMA production by the intestinal microbiota bacteria	Targeting bacterial TMA-production.
3,3-dimethyl-1-butanol [38]	Inhibition of TMA formation through inhibition of microbial TMA lyases	Other inhibitory mechanisms e.g.,: changes in microbial taxa or inhibition of foam cell formation and atherosclerotic lesion
FMO3 enzyme inhibition [57]	Prevented the oxidization of TMA to TMAO	It has other effects on the regulation of both lipid metabolism and inflammation
